# Vitamin C Treatment Rescues Prelamin A-Induced Premature Senescence of Subchondral Bone Mesenchymal Stem Cells

**DOI:** 10.1155/2020/3150716

**Published:** 2020-04-03

**Authors:** Yan-Nv Qu, Li Zhang, Ting Wang, He-Yang Zhang, Ze-Ji Yang, Fang-Fang Yuan, Yan Wang, Si-Wei Li, Xiao-Xia Jiang, Xiao-Hua Xie

**Affiliations:** ^1^Chinese PLA General Hospital, 28 Fuxing Road, Haidian District, Beijing 100853, China; ^2^Department of Neural Engineering and Biological Interdisciplinary Studies, Institute of Military Cognition and Brain Sciences, Academy of Military Medical Sciences, 27 Taiping Road, Haidian District, Beijing 100850, China

## Abstract

Aging is a predominant risk factor for many chronic conditions. Stem cell dysfunction plays a pivotal role in the aging process. Prelamin A, an abnormal processed form of the nuclear lamina protein lamin A, has been reported to trigger premature senescence. However, the mechanism driving stem cell dysfunction is still unclear. In this study, we found that while passaging subchondral bone mesenchymal stem cells (SCB-MSCs) *in vitro*, prelamin A accumulation occurred concomitantly with an increase in senescence-associated *β*-galactosidase (SA-*β*-Gal) expression. Unlike their counterparts, SCB-MSCs with prelamin A overexpression (MSC/PLA) demonstrated decreased proliferation, osteogenesis, and adipogenesis but increased production of inflammatory factors. In a hind-limb ischemia model, MSC/PLA also exhibited compromised therapy effect. Further investigation showed that exogenous prelamin A triggered abnormal nuclear morphology, DNA and shelterin complex damage, cell cycle retardation, and eventually cell senescence. Changes in gene expression profile were also verified by microarray assay. Interestingly, we found that ascorbic acid or vitamin C (VC) treatment could inhibit prelamin A expression in MSC/PLA and partially reverse the premature aging in MSC/PLA, with reduced secretion of inflammatory factors and cell cycle arrest and resistance to apoptosis. Importantly, after VC treatment, MSC/PLA showed enhanced therapy effect in the hind-limb ischemia model. In conclusion, prelamin A can accelerate SCB-MSC premature senescence by inducing DNA damage. VC can be a potential therapeutic reagent for prelamin A-induced aging defects in MSCs.

## 1. Introduction

Aging is defined as a progressive deterioration of an organism's physiological functions. Aging is a predominant risk factor for many chronic conditions, including atherosclerosis, cancer, and neurodegenerative and metabolic syndromes, which consequently increases susceptibility to death [1]. Stem cells retain the capacity to differentiate into the cell types of their constituent tissues and contribute to regeneration and homeostasis [2]. Stem cell exhaustion is associated with the decline in the regenerative potential capacity linked to the accumulation of age-associated damage [3]. Recent studies have shown that targeting cellular senescence prevents age-related disease in animal models [4–8]. Therefore, it is crucial to understand the mechanisms of stem cell senescence in order to prevent aging.

MSCs, owing to their excellent regenerative and immunomodulatory effects, have been widely used in cell therapy for the repair and regeneration of tissues and organs. However, recent studies have shown that the quality of MSCs declines with age [9, 10]. Subculturing MSCs *in vitro*, to obtain therapeutic doses under traditional culture conditions, has been shown to increase the possibility of cell senescence. Previous studies have demonstrated that pathological accumulation of some proteins can cause severe alterations in nuclear organization, hampering the normal functions of the cells and ultimately leading to premature aging [11]. Lamin A, a key organizer of the mammalian cell nucleus [12], has been mechanistically linked to most of the universal hallmarks of aging [13]. Accumulation of prelamin A (PLA), a precursor of lamin A, has been reported to induce defects in nuclear structures, leading to premature cell senescence in physiological and pathological aging [14–17]. Though some studies imply that prelamin A can induce senescence in hMSCs [18, 19], the effect of prelamin A in MSCs isolated from senior donors (50-60 years old) has not been evaluated.

In this study, we isolated SCB-MSCs from the abandoned tibial plateau of patients undergoing knee replacement surgery (aged from 50-60 years) and cultured them to analyze the aging characteristics. We found accumulation of prelamin A during the culturing of SCB-MSCs *in vitro*. SCB-MSCs transduced with adenovirus containing prelamin A demonstrated abnormal nuclear morphology and inferior multidifferentiation potential, higher DNA damage, showing most cell senescence characteristics. Importantly, our data demonstrated that a high dose of ascorbic acid or vitamin C (VC) treatment could inhibit prelamin A expression and partially reversed the premature aging of MSCs, indicating that VC may be a potential therapy for prelamin A-induced aging in MSCs.

## 2. Materials and Methods

### 2.1. Isolation of SCB-MSCs and Cell Culture

MSCs were isolated from the normal side of abandoned tibial plateau of patients undergoing knee replacement surgery (aged from 50-60 years, Table S1). To isolate MSCs, the subchondral bone fragments of the abandoned tibial plateaus were carefully excised with scissors and were collected using forceps. Subchondral bone fragments were digested with 1 mg/ml (*w*/*v*) collagenase II (Gibco) at 37°C for 20 min. The digestion medium was discarded, and the enzyme-treated subchondral bone fragments were seeded on plastic culture dishes (T25) in the presence of *α*-MEM (Gibco), supplemented with 10% (*v*/*v*) fetal bovine serum (FBS), 100 U/ml penicillin, and 100 *μ*g/ml streptomycin. The culture medium was changed on the third day of culture, and the tissue debris was maintained to allow more MSCs to grow. Cells were used between the 3^rd^ and 5^th^ passages.

### 2.2. Cell Transfection and Treatment

Primary SCB-MSCs (passage 3) were plated on a 6-well plate at a cell density of 1.5 × 10^5^ cells/well. At 70% to 80% confluence, the cells were infected with flag-tagged recombinant adenoviruses containing prelamin A (PLA) or flag-tagged recombinant adenoviruses containing green fluorescent protein (GFP) at the MOI of 30-50. Five days postinfection, the cells were ready for further tests.

After cell transfection, culture medium with VC (sigma, A8960) was changed every other day for seven days. The relative cell proliferative abilities of SCB-MSCs (passage 4) treated with different concentrations were analyzed at day 7.

For proliferation studies, the cells were seeded at a cell density of 1.5 × 10^5^ cells/well and cells were counted in triplicate on each passage until proliferation ceased. The population doubling time (PDT) was calculated using the formula Td = (T 2 − T 1)^∗^ log(2)/Log(P 2/P 1) [20], where Td is doubling time, P1 is the number of cells at time T1, and P2 is the number of cells at time T2.

### 2.3. Western Blot

Cells were lysed in Laemmli sample buffer (BioRad), and the amount of protein present in the lysed sample was quantified using BCA Protein Assay (BioRad). Equal amounts of proteins were resolved using a 12% SDS-PAGE gel and transferred to a PVDF membrane (Life Science). After blocking, the membrane was incubated sequentially with the primary antibodies and secondary peroxidase-conjugated antibodies. Immobilon Western Chemiluminescent HRP (Millipore) was used for protein visualization using a ChemiDoc (BioRad). Bands were quantified using ImageJ software, and the results were expressed relative to the control.

Antibodies used for Western blot analysis or immunofluorescence labeling were anti-prelamin A, rabbit polyclonal (sc-518013 C-3, Santa Cruz), raised specifically against an epitope mapping between amino acids 580 and 601 near the **C**-terminus of lamin A of human origin but not the SIM sequence.p16 (#80772), p21 (#2947), p53 (#2524), phosphor-H2AX (#2577), and 53BP1 (#4937) are all from Cell Signaling Technology and anti-*β*-actin (sc-47778, Santa Cruz).

### 2.4. RNA Isolation and Quantitative Reverse Transcription Polymerase Chain Reaction (RT-PCR)

Total RNA was extracted from the cells using Trizol reagent (Ambion, Life technologies), and 1 *μ*g of RNA was reverse-transcribed using the Goldenstar RT6 cDNA Synthesis Mix (Takara, Kyoto, Japan) according to the manufacturer's instructions. SYBR Pre-mix ExTaq (Takara, Kyoto, Japan) was used for real-time PCR with the FTC-3000P (Funglyn Biotech Inc) real-time PCR system following the manufacturer's instructions; primer sequences are listed in Table S2.

### 2.5. Flow Cytometry Analysis

The human MSCs were identified by staining against CD73 (Ecto-5′-nucleotidase), CD90 (Thy-1) and CD105 (Endoglin), CD44- (CSPG8) positive cells and negative to CD31 (PECAM1), and CD45 (PTPRC). SCB-MSCs were collected and washed twice by PBS. Cells were incubated with primary antibody diluted in PBS for 30 min at 4°C temperature and analyzed by flow cytometry (BD FACS Aria IIIu). Mouse anti-human CD105, CD90, CD73, CD44, CD45, CD146 (MCAM), and CD31 were obtained from BioLegend. The isotype-matched mouse IgG1-FITC, IgG1-PE, IgG1-APC, and IgG1-percp were used as negative controls. Data, acquired and processed from 8,000 events, were analyzed using FlowJo software.

### 2.6. Senescence-Associated *β*-Galactosidase (SA-*β*-Gal) Staining

SA-*β*-Gal staining was performed using X-GAL kit (#9860S, Cell Signaling Technology), following the manufacturer's protocols. Briefly, cultured cells were washed in PBS and fixed at room temperature for 30 min with fixing solution. Fixed cells were stained with fresh staining solution for SA-*β*-Gal activity at pH 6, 37°C overnight, and positive cells were quantified using ImageJ software.

### 2.7. Multidifferentiation of SCB-MSCs

*In vitro* multidifferentiation potential of SCB-MSCs was tested as described previously with minor modifications [21]. Briefly, for osteogenic differentiation, SCB-MSCs at passage 3 were seeded on 24-well culture plates, cultured in presence of osteogenic induction medium for 14 days. The osteogenic induction medium consisted of culture medium, 0.1 *μ*M dexamethasone, 10 mM *β*-glycerophosphate, and 50 *μ*M ascorbic acid (Sigma-Aldrich). The osteogenic differentiation of MSCs was assayed by in situ alkaline phosphatase (ALP) staining using a commercial kit (Sigma-Aldrich) at day 7 and Alizarin red (Sigma-Aldrich) staining at day 14.

For adipose differentiation, SCB-MSCs were cultured in the presence of adipogenic induction medium (#05412, Stemcell) for 14 days. Oil Red O (Sigma) staining of SCB-MSC-derived adipocytes was performed to stain lipid droplets following a standard protocol with some modifications [22].

### 2.8. Immunofluorescence

SCB-MSCs at passage 3 were seeded on glass coverslips placed in 24-well culture plates and fixed in 4% paraformaldehyde (PFA). Cells were examined by confocal fluorescence microscopy (Leica SP8, Confocal microscope). Immunofluorescence was performed using the following antibodies: prelamin A (sc-518013 C-3, Santa Cruz), phospho-histone H2AX (#2577, Cell Signaling Technology), 53BP1 (#4937, Cell Signaling Technology), Ki67 (ab15580, Abcam), calponin 1 (ab46794, Abcam), alpha smooth muscle actin (ab124964, Abcam), mCD31(#14-0311-82, eBioscence), and *β*-actin (sc-47778,Santa Cruz). 4′,6-diamidino-2-phenylindole (DAPI) (zli-9557, Zhongshan) was used to counterstain cell nuclei. Around 100 randomly selected cells were analyzed.

### 2.9. Cell Cycle and Apoptosis Detection

Cells were trypsinized and washed 3 times in PBS, then resuspended in PBS containing 1% FBS and fixed in 75% ice-cold ethanol overnight at -20°C. After addition of propidium iodide (PI) and RNase, the cells were incubated for 30 minutes at 37°C, following which, they were sorted using flow cytometry. For Annexin V/7AAD staining, cells were processed according to the manufacturer's instructions (BioLegend).

### 2.10. Murine Model of Hind-Limb Ischemia and Cell Transplantation

C57BL/6 mice (male 8-12 w) were selected to induce hind-limb ischemia. Mice were anaesthetized with avertin (240 mg/kg, Sigma) by intraperitoneal injection. Right hind-limb ischemia was induced as previously described with minor modifications [22, 23]. The femoral vessels (artery and vein) were dissected and separated from the nerve. Using a 6.0 nonabsorbable polypropylene suture, the distal external iliac artery and vein (both deep femoral artery and superficial femoral artery) were ligated, excised from femoral proximal origin, deep femoral artery, and superficial femoral artery. Randomized, double-blinded study design was used. Mice were randomly assigned to the following experimental groups: PBS group, MSC/GFP group, MSC/PLA group, and MSC/PLA+VC group (MSC/PLA pretreated with 200 *μ*M VC for 7 days). Before injection, cells were washed twice in PBS, then counted. Viability was determined by Trypan blue staining and was above 90 ± 6%. Cells were resuspended in PBS. A total of 3 × 10^5^ of cells (MSC/GFP, MSC/PLA, or MSC/PLA+VC) or PBS (PBS) in a volume of 100 *μ*l were injected intramuscularly into 4 sites of the tibialis anterior muscle. Animals were carefully observed every 15-20 minutes to make sure all animals recover well from the antisedative.

### 2.11. Blood Flow Analysis

Blood flow perfusion was assessed as described previously [23]. Tissue perfusion of the hind limbs was assessed with a Laser-Doppler perfusion imager (Perimed AB) on days 0, 7, and 14 after ligation. The digital color-coded images were analyzed to quantify blood flow in the region from the external iliac artery just above the inguinal ligament, to the toe. Measures were taken under anesthesia with avertin, room temperature 26°C. Ratios of the ischemic (right)/normal (left) limb blood flow were used to express the results. The mean and the standard deviation were calculated for each group.

Clinical recovery: physiological and histological examination and blood flow perfusion analysis were performed. The surgery and the evaluation of the results were independently performed by two individuals.

Physiological status of the ischemic limbs was evaluated in three levels: limb loss, limb salvage, and foot necrosis. Limb loss was defined as the loss of limb from the knee or above. Limb salvage means the retention of limb without foot necrosis. Foot necrosis included loss of the foot and toes.

### 2.12. Tissue Preparation and Immune-Histological Analysis

The gastrocnemius muscles of each mouse were harvested at day 14 or day 21 for histological analysis. The muscles were fixed in 4% PFA for staining with hematoxylin, eosin, and Masson's trichrome. For immunofluorescence, tissues were embedded in optimal cutting temperature (OCT) compound and frozen sections of 40 *μ*m thickness were mounted on superfrost slides. Sequentially, immunofluorescence staining was performed; slides were examined by confocal fluorescence microscopy (Leica SP8, Confocal microscope).

### 2.13. Microarray Information and Data Assay

The Agilent Human lncRNA Microarray 2018 (4^∗^180 k, Design ID: 085630) was used in this experiment, and data analysis was performed by OE Biotechnology Co., Ltd. (Shanghai, China).

Total RNA was quantified using NanoDrop ND-2000 (Thermo Scientific), and the RNA integrity was assessed using Agilent Bioanalyzer 2100 (Agilent Technologies). The sample labeling, microarray hybridization, and washing were performed according to the manufacturer's protocols. Briefly, total RNA was transcribed to double-strand cDNA; then, cRNA was synthesized and labeled with Cyanine-3-CTP. The labeled cRNAs were hybridized on the microarray. After washing, the arrays were scanned by the Agilent Scanner G2505C (Agilent Technologies).

Feature Extraction software (version10.7.1.1, Agilent Technologies) was used to analyze array images to get the raw data. Genespring (version 14.8, Agilent Technologies) were employed to finish the basic analysis with the raw data. Differentially expressed genes were then identified through fold change; *P* value calculated using Student's *t* test. The threshold set for upregulated and downregulated genes was a fold change ≥2.0 and a *P* value ≤ 0.05. Afterwards, GO analysis and KEGG analysis were performed to determine the roles of these differentially expressed mRNAs. Finally, hierarchical clustering was performed to display the distinguishable expression pattern of the genes among samples. The Signal2Noise method in the gene set enrichment analysis (GSEA) was used to analyze the data.

## 3. Statistical Analysis

Quantitative variables are presented as mean ± SD. Comparison of continuous variables was performed using unpaired Student's *t* test; *χ*^2^ test was used for categorical variables. For multiple comparisons, two-way ANOVA was performed, followed by Bonferroni multiple comparison. Statistical analyses were performed using the GraphPad software. A value of *P* < 0.05 was considered statistically significant.

## 4. Results

### 4.1. Prelamin A Overexpression Triggered SCB-MSC Premature Senescent Phenotype and Attenuated the Proliferation Capacity

**Figure 1:**
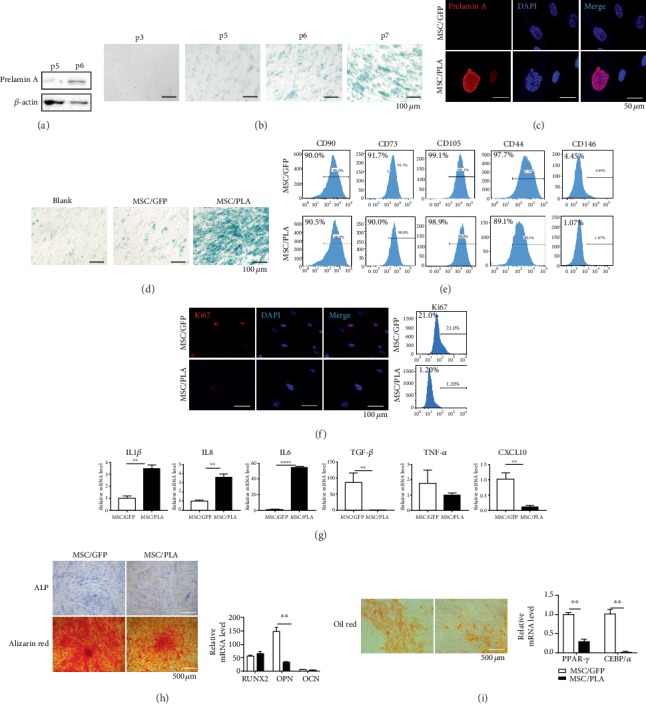
Prelamin A accumulation triggered premature senescent phenotype and reduced multidifferentiation potential. (a) Prelamin A accumulated after *in vitro* subculturing. (b) SA-*β*-Gal activity increased after passages. Scale bar: 100 *μ*m. (c) Representative confocal images showed nuclear membrane abnormalities, nuclear enlargement, and morphology defects. Scale bar: 50 *μ*m. (d) Prelamin A triggered the increase activity of SA-*β*-Gal. Scale bar: 100 *μ*m. (e) Immunophenotypic features of stem cell markers in MSC/GFP and MSC/PLA. (f) Representative confocal images (left) and flow cytometry (right) demonstrated the expression of Ki67 in MSC/GFP and MSC/PLA. Scale bar: 100 *μ*m. (g) Comparison of mRNA expression levels of cytokines between MSC/GFP and MSC/PLA (^∗∗^*P* < 0.001, ^∗∗∗∗^*P* < 0.0001). (h) ALP staining (top, left) and alizarin red (bottom, left) demonstrated reduced osteogenic potential in MSC/PLA. Scale bar: 500 *μ*m. Comparison of mRNA expression levels (right) of osteogenic related genes (Runx-2 and OPN, OCN). Oil red staining (I, left) and the mRNA expression levels of PPAR-*γ* and CEBP/*α* (I, right) demonstrated adipogenic characteristics in the two groups. *n* = 3 independent experiments (^∗∗^*P* < 0.001).

SCB-MSCs were obtained from 5 different patients (both men and women, aged from 50 to 60 years, Table S1). Seven days after primary culture, fibroblast-like cells migrated from the SCB fragments and adhered to the plate (Fig. S1(a)). SCB-MSCs exhibited adult stem cell characteristics, positive for CD73, CD90, CD105, and CD44, but negative for the hematopoietic markers CD45 and CD31 [24] (Fig. S1(b)), and multidifferentiation potential (Fig. S1 D). However, prelamin A upregulation was detected in later passages (passages 5 and 6) of SCB-MSCs (Figure 1(a)). In addition, SA-*β*-Gal activity increased and proliferation decreased in later passages of SCB-MSCs (Figures 1(b) and S1(c)), suggesting a relationship between prelamin A and cell senescence. Considering all these data, passages 3 to 5 SCB-MSCs were chosen to perform the following experiments. To determine whether prelamin A induces senescence in SCB-MSCs, flag-tagged recombinant adenoviruses containing GFP-prelamin A were used (Fig. S1(e) and S1(f)). Immunostaining showed that these SCB-MSCs accumulated nuclear prelamin A and showed abnormal nuclear morphology, compared with SCB-MSCs transduced with GFP (MSC/GFP) (Figure 1(c)). The increase in SA-*β*-Gal activity in prelamin A-overexpressing SCB-MSCs (MSC/PLA) indicated increased cellular senescence (Figure 1(d)). There were no significant changes in the presence of mesenchymal markers, including CD73, CD90, and CD44 (Figure 1(e)), between MSC/GFP and MSC/PLA. However, self-renewal ability, assayed by Ki67 staining, decreased dramatically (Figure 1(f)), suggesting attenuated proliferation.

Senescent cells secrete proinflammatory cytokines, chemokines, and proteases, termed the senescence-associated secretory phenotype (SASP), which was also detected in prelamin A accumulated SCB-MSCs. Previous studies have shown that inflammatory factors, such as IL1 *β*, IL6, IL8, and MCP-1, are abundantly secreted by vascular smooth muscle cell (VSMC) in response to prelamin A-induced persistent DNA damage [25]. As expected, the mRNA levels of IL1 *β*, IL6, and IL8 were also upregulated markedly with a decrease in TGF-*β* and CXCL 10 [26] (Figure 1(g)).

MSCs have multiple differentiation potentials, including osteogenic, adipogenic, and chondrogenic lineages. First, a significant decrease was observed in ALP activity and alizarin red staining, suggesting that the osteogenic differentiation potential in MSC/PLA was weak compared to that in MSC/GFP after 7 and 14 days of induction (Figure 1(h)). Second, there was a decrease in adipogenesis induction as suggested by oil red o staining (Figure 1(i)).

### 4.2. Prelamin A Overexpression Attenuated the Therapy Effect of SCB-MSCs in Hind-Limb Ischemia

**Figure 2:**
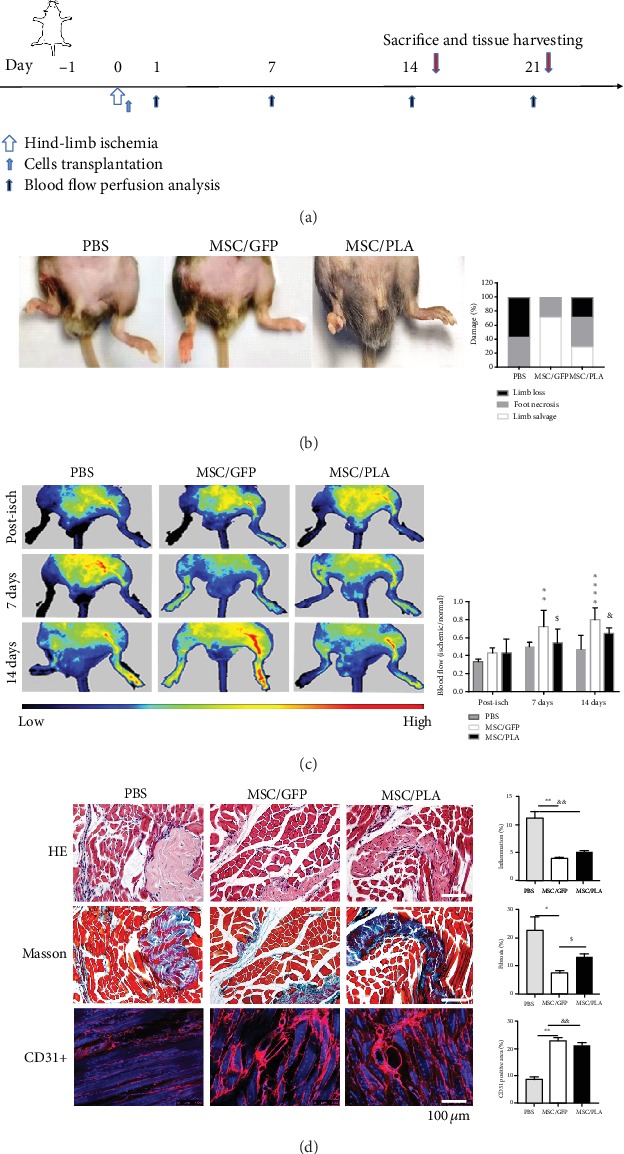
Transplantation of MSC/PLA attenuated the rescue effect of SCB-MSCs in hind-limb ischemia. (a) Experimental paradigm for transplantation study. (b) Representative pictures of limb survival of PBS, MSC/GFP, and MSC/PLA groups. (c) Representative Laser-Doppler flow images (left) post-isch, and at days 0, 7, and 14 of PBS, MSC/GFP, and MSC/PLA, respectively. Quantitative evaluation of blood flow (right) expressed as a ratio of ischemic to normal limb demonstrated a significant increase of limb blood perfusion in the MSC/GFP group, compared with the PBS and MSC/PLA groups. (d) Histological analysis on cross sections in the PBS, MSC/GFP, or MSC/PLA groups in ischemic legs. Hematoxylin and eosin staining (top, left) and quantification (top, right) for infiltration of numerous granulocytes and neutrophils. Scale bar: 50 *μ*m. Masson trichrome staining (middle, left) and quantification (middle, right) visualized fibrosis in hind-limb ischemia. Visualization of neovascularization by immunofluorescence images (bottom, left) and quantification (bottom, right) with CD31 labeling. Scar bar:100 *μ*m (MSC/GFP vs. PBS: ^∗^*P* < 0.05, ^∗∗^*P* < 0.001, ^∗∗∗∗^*P* < 0.0001; MSC/PLA vs. PBS: ^&^*P* < 0.05, ^&&^*P* < 0.001, MSC/GFP vs. MSC/PLA: ^$^*P* < 0.05).

Hind-limb ischemia was used to assay the functional competence of SCB-MSCs accumulated prelamin A *in vivo*, based on the ability of MSCs of improving vascular circulation after transplantation [27]. First, ischemia was induced in mouse right limb by ligation of the femoral artery and its branches in the right hind limb of C57BL/6 mice [28]. Then, control (PBS), MSC/GFP, and MSC/PLA were transplanted though intramuscular injection of into the tibialis anterior muscle, *n* = 14 per group. Three levels of the therapy effect, limb salvage, foot necrosis, and limb loss, were evaluated among the three groups on day 0 (after surgery), day 7, and day 14 (Figure 2(a)). At the end of therapy, 14 days after transplantation, 8 (57.1%) had limb loss and 6 (42.8%) demonstrated extensive foot necrosis in the PBS group. In the MSC/GFP group, limb salvage was observed in 8 (71.2%) and 4 (28.4%) displayed mild to moderate necrosis from toe to knee. In contrast, limb salvage, 4 (28.5%), pulled down sharply in the MSC/PLA group, with 6 (42.8%) foot necrosis and 4 (28.5%) limb loss (Figure 2(b) left and right).

Laser-Doppler flow imaging of hind limb was performed to assess the impact of cell transplantation on blood perfusion at day 0 (after surgery), day 7, and day 14. Repeated-measures ANOVA analysis of blood perfusion in the three groups demonstrated a significant difference (*P* < 0.001) at day 14. Laser Doppler images showed a low ratio of blood perfusion persistent over 14 days in the PBS group, indicating the development of severe hind-limb ischemia (Figure 2(c) left and right; *P* = 0.0542, day 0 versus 14). In the MSC/GFP and MSC/PLA transplantation groups, blood perfusion was gradually recovered over 14 days with no significant difference (*P* = 0.0937). However, the MSC/GFP group demonstrated significant increase in blood perfusion at day 7 unlike MSC/PLA (*P* < 0.05), suggesting delayed and inferior neovascularization in the MSC/PLA group to the MSC/GFP group (Figure 2(c) left and right).

Further evaluation of therapy effect was performed using histological techniques using 20 slices from inconsecutive frozen sections from seven mice in each group. Histological analysis of ischemic limbs at day 14 after transplantation showed massive muscle degeneration, granulocytes and neutrophils' infiltration by hematoxylin and eosin staining,and replacement of fibrous connective tissue, demonstrated by Masson's trichrome staining (Figure 2(d) top and middle). Beyond the obvious replacement of fibrosis, infiltration of numerous granulocytes and neutrophils were found wildly in the PBS group. The MSC/GFP and MSC/PLA groups displayed ameliorated fibrosis and muscle degeneration and were partly rescued, following the less infiltration of numerous granulocytes and neutrophils in hind-limb ischemia (Figure 2(d) top and middle). The MSC/PLA group showed more fibrosis than the MSC/GFP group (*P* = 0.0183). Significantly attenuated inflammation was observed in MSC/PLA-treated limbs compared with the PBS group (*P* = 0.0176).

Immunofluorescent images of mCD31-positive vessels showed neovascularization. The capillary densities were measured in two different sections of four distinct anatomic areas in each specimen, using ImageJ. At day 14, anti-mCD31 staining revealed overall neovascularization that was not significantly different between the MSC/GFP and MSC/PLA groups, which was superior to the PBS group (Figure 2(c) bottom left). Quantitative measurement was reported as percent of positively stained mCD31 cells in the MSC/GFP and MSC/PLA groups, visible neovascularization in hind-limb ischemia, compared with the PBS group (Figure 2(c) bottom, right, *P* < 0.001).

### 4.3. Prelamin A-Induced DNA Damage and Unstable Expression of Shelterin Complex in SCB-MSCs

**Figure 3:**
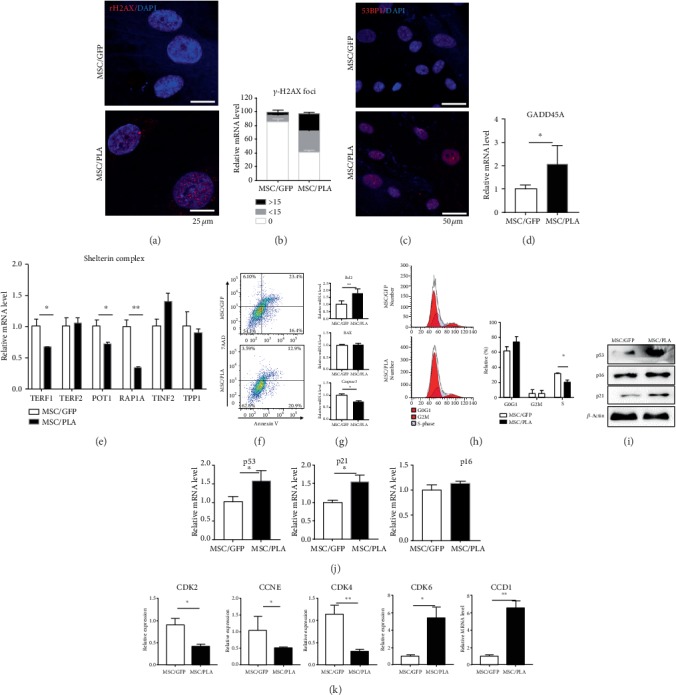
Prelamin A accumulation triggered SCB-MSCs and increased DNA damage, apoptosis resistance, and cell cycle arrest. Representative confocal images of (a) *γ*-H2AX staining and (b) quantification of *γ*-H2AX foci. Blue: DAPI; red: *γ*-H2AX. Scale bar: 25 *μ*m. At least 100 nuclei were analyzed per sample. *χ*^2^ was used for statistical significance. (c) Representative confocal images of 53BP1 in MSC/GFP and MSC/PLA. Blue: DAPI; red: 53BP1. Scale bar: 50 *μ*m. (d) Remarkable increase of GADD45A mRNA level (^∗^*P* < 0.05). (e) Comparison of mRNA expression levels of Shelterin complex (POT1, RAP1A, TERF1, TERF2, TPP1, and TINF2) (^∗^*P* < 0.05, ^∗∗^*P* < 0.01). (f) The apoptotic cells were decreased in SCB-MSC overexpressing prelamin A using Annexin V/7AAD. (g) Quantitative RT-PCR showed the downregulation of caspase 3 and the significant elevation in the mRNA level of Bcl2 (^∗^*P* < 0.05, ^∗∗^*P* < 0.01). (h) Flow cytometry analysis showed plunged S phase in the MSC/PLA, compared with the MSC/GFP (^∗^*P* < 0.05). (i) Protein levels of p21 and p53 significantly increased in prelamin A-overexpressed SCB-MSCs. (j) Quantitative RT-PCR verified the upregulation of p53 and p21 in MSC/PLA (^∗^*P* < 0.05). (k) The downregulated CDK2 and CCNE in the MSC/PLA in mRNA level demonstrated the block in G1/S checkpoint (^∗^*P* < 0.05, ^∗∗^*P* < 0.01).

Since the proliferation and potential function waning, we investigated the possible damage caused by the overexpression of prelamin A. Abnormal nuclear morphology is known to trigger DNA damage in VSMCs [29], phosphorylate H2AX at Ser139 forming *γ*-H2AX which leads to recruitment of DNA repair proteins, such as 53BP1 and BRCA1 [30]. Visualization of *γ*-H2AX is possible using immunofluorescence assays. As expected, the number of *γ*-H2AX foci increased in MSC/PLA when comparing with MSC/GFP (Figures 3(a) and 3(b)). Previous studies using *γ*-irradiation mouse embryonic fibroblast (MEF) from Zmpste 24 -/- mice, which are deficient in lamin A processing enzyme, have shown that Pan-nuclear 53BP1 protein was redistributed into distinct foci [31]; pan-nuclear 53BP1 protein was also detected in MSC/PLA (Figure 3(c)). At the same time, mRNA levels of growth arrest and DNA damage-inducible 45 (GADD45A) were upregulated (Figure 3(d)).

Mammalian telomeres are associated with shelterin, a protein complex that protect DNA ends from being recognized as double strand breaks thereby limiting the trigger for a DNA damage response [32, 33]. Proteins comprising the shelterin complex are telomere protection protein 1 (TPP1), telomere repeat factor (TERF) 1, TERF2, TERF1 interacting nuclear factor 2 (TINF2), repressor activator protein 1A(RAP1A), and protection of telomere protein 1 (POT1) [33]. The important role for telomere–protein complex formation is underscored by the high frequency of telomere end-fusions and inappropriate recombination that occur in the absence of telomere protection proteins [34]. The levels of three of the six components in telomere attrition associated shelterin complex, POT1, RAP1A, and TERF1 decreased, indirectly suggesting telomere attrition (Figure 3(e)).

Activation of DNA damage response including formation of DNA damage foci containing activated H2AX (*γ*-H2AX) at uncapped telomeres or at persistent DNA strand breaks is the major trigger for cell senescence [35]. DNA damage triggers the DNA repair machinery, apoptosis, or senescence depending on the extent of damage and the physiological context. P53 and its downstream targets are involved in DNA damage repair [36] and genome integrity; apoptosis or senescence is triggered if the repair is incomplete. Contrary to our expectations, flow cytometry analysis showed lower positive expression of Annexin V in MSC/PLA, compared to MSC/GFP (Figure 3(f)). After prelamin A overexpression, SCB-MSCs exhibited a measurable downregulation of caspase 3, significant elevation in the mRNA level in Bcl2, and increased levels of the apoptotic Bcl2/BAX ratio (Figure 3(g)), suggesting resistance to apoptosis.

To understand the mechanism behind the decreased proliferation potential of MSC/PLA, cell cycle and senescence markers were assayed in MSC/GFP and MSC/PLA. Flow cytometry of propidium iodide- (PI-) stained cells showed significant retardation in S stage, suggesting that cell cycle was blocked at G1/S checkpoint (Figure 3(h)). The overexpression of prelamin A in SCB-MSCs increased the expression of p53 and p21 proteins, without significant change in p16, which was also validated using quantitative RT-PCR (Figures 3(i) and 3(j)). Cyclin-dependent kinases and cyclins were evaluated by quantitative RT-PCR. Both CDK2 and CCNE decreased sharply in MSC/PLA, inducing a block in G1/S checkpoint (Figure 3(k)).

### 4.4. Microarray Analysis Reveals Changes in Gene Expression Profile in MSC/PLA

**Figure 4:**
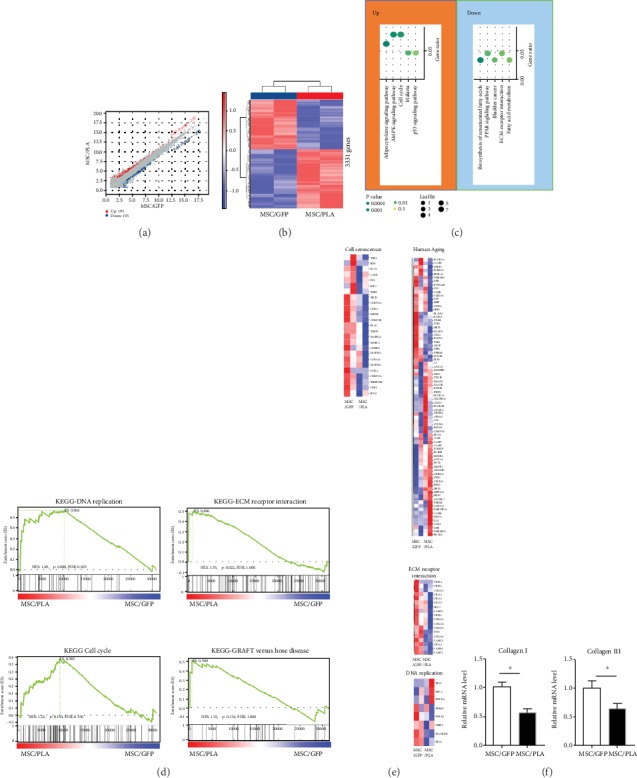
Gene expression profile analysis of MSC/GFP and MSC/PLA. (a) Volcano plot showing the number of upregulated and downregulated genes on prelamin A overexpression. (b) Heatmap illustrating differentially expressed genes between replicates of MSC/GFP and MSC/PLA (passage 5). (c) KEGG enrichment analysis of upregulated genes in MSC/PLA. (d) Gene set enrichment analysis (GSEA) plots showing representative gene terms upregulated by prelamin A overexpression. (e) Heatmaps showing the transcriptional levels of genes enriched in various gene terms. (f) Quantitative RT-PCR verified the decreased expression of collagen I and collagen III in MSC/PLA (^∗^*P* < 0.05).

In order to investigate the whole transcriptome expression changes during prelamin A accumulation, microarray analysis comparing SCB-MSCs infected with control or prelamin A adenovirus was used. Microarray identified significant changes in the levels of 386 mRNA (193 upregulated, 193 downregulated), according to previous 2 principal components (Figures 4(a) and 4(b), Table S3). KEGG analysis revealed that the upregulated and downregulated pathways in MSC/PLA were enriched in pathways. Top 3 upregulated enriched pathways adipocytokine signaling pathway (*P*value: 0.0032), AMPK signaling pathway (*P*value: 0.0043) and cell cycle (*P*value: 0.0047). On the other hand, the top 5 downregulated pathways showing enrichment were biosynthesis of unsaturated fatty acids (*P* value: 0.002), PPAR signaling pathway (pvalue:0.0108), and extracellular matrix (ECM)-receptor interaction (*P* value: 0.0168) (Figure 4(c)). Consistently, gene set enrichment analysis (GSEA) data revealed that MSC/PLA were enriched in KEGG pathways including ECM receptor, DNA replication, graft versus host disease, and cell cycle. These results indicate alterations in DNA replication, cell cycle, and amplified inflammatory factors, consistent with our findings above (Figure 4(d)). Heatmaps show the transcriptional levels of genes enriched in DNA replication, cell senescence, ECM receptor interaction, and human aging, which verified the genomic abnormality in MSC/PLA (Figure 4(e)). In addition, we found that collagen I and collagen III plunged down in MSC/PLA, thus validating the observed change in ECM (Figure 4(f)).

### 4.5. Vitamin C Alleviated Premature Senescence in MSC/PLA

Previous studies on *C. elegans*, WRN-deficient human mesenchymal stem cells, and mouse models have shown that a continuous long-term treatment of WS individuals with high doses of VC is a promising therapeutic approach [37–39]. However, the effect of VC on cell senescence triggered by prelamin A accumulation has not yet been studied. We investigated the effects of VC treatment in prelamin A overexpressed SCB-MSCs.

**Figure 5:**
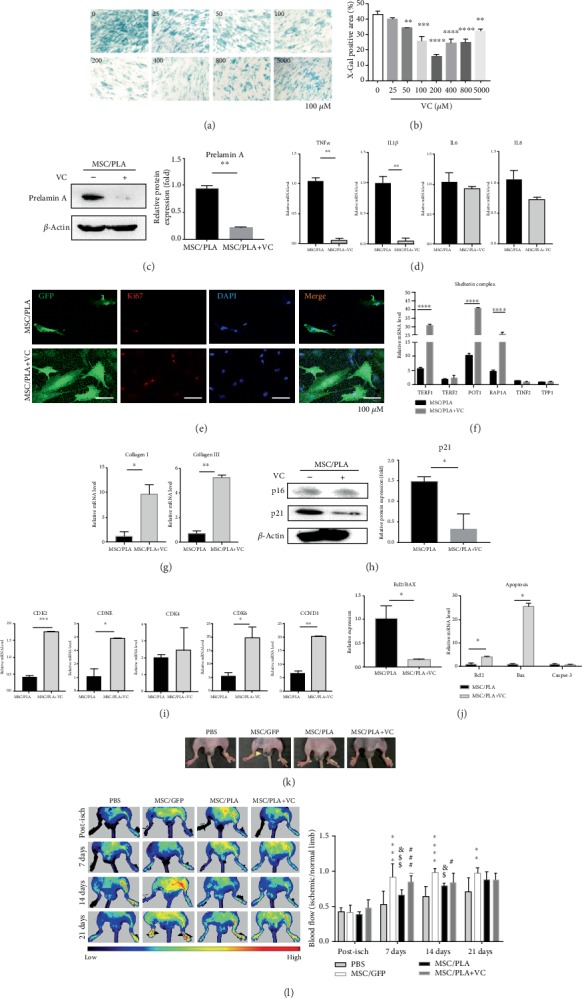
VC alleviated premature senescence in MSC/PLA. Representative images (a) and quantification (b) of SA-*β*-Gal-positive cells in MSC/PLA treated with different concentrations of VC. Data are represented as mean ± SEM, ^∗∗^*P* < 0.01, ^∗∗∗^*P* < 0.005, ^∗∗∗∗^*P* < 0.0001, *n* ≥ 3. The significant differences between means were calculated by ANOVA. Scale bar: 100 *μ*m. (c) Immunoblots demonstrated the significant decreased prelamin A in protein level after VC treatment. (d) The decrease in expression of TNF*α* and IL1*β* in mRNA level compared MSC/PLA with or without VC treatment (^∗∗^*P* < 0.01). (e) Representative confocal images of the expression of Ki67 in MSC/PLA treated with or without VC treatment. (f) VC restored decreased expression of TERF1, POT1, and RAP1A in MSC/PLA (^∗∗∗∗^*P* < 0.001). (g) Quantitative RT-PCR illuminated the increase in expression of collagen I and collagen III in MSC/PLA treated with VC (^∗^*P* < 0.05, ^∗∗^*P* < 0.01). (h) P21 downregulated after VC treatment in MSC/PLA (^∗^*P* < 0.05). (i) VC rescued the expression of CDK2 and CCNE at the mRNA level and partly ameliorated cell cycle arrest in MSC/PLA (^∗^*P* < 0.05, ^∗∗^*P* < 0.01). (j) Quantitative RT-PCR illuminated decreased levels of the apoptotic Bcl2/BAX ratio after VC treatment (^∗^*P* < 0.05). (k) Representative pictures of limb survival of PBS, MSC/GFP, MSC/PLA, and MSC/PLA+VC groups. (l) Representative Laser-Doppler flow images (left) postischemia at days 0, 7, 14, and 21 of PBS, MSC/GFP, MSC/PLA, and MSC/PLA+VC. Quantitative evaluation of blood flow (right) expressed as a ratio of ischemic to normal limb demonstrated significantly enhanced limb blood perfusion in MSC/GFP compared with PBS and MSC/PLA (MSC/GFP vs. PBS, ^∗∗^*P* < 0.01, ^∗∗∗∗^*P* < 0.0001; MSC/PLA vs. PBS, ^&^*P* < 0.05; MSC/GFP vs. MSC/PLA, ^$^*P* < 0.05; MSC/PLA vs. MSC/PLA+VC ^~^*P* < 0.05; MSC/PLA+VC vs. PBS, ^#^*P* < 0.05).

After 7 days of pretreatment with VC, a dose-dependent decrease in SA-*β*-Gal-positive cells was observed (Figure 5(a)). The efficacy of VC on suppressing senescence was very prominent at 50 *μ*mol/L, relative to MSC/PLA (*P* = 0.0083) (Figure 5(b)). The efficacy of VC treatment increased sharply in a dose-dependent manner and reached saturation at 200 *μ*mol/L. The SA-*β*-Gal-positive cells increased with the increase in VC dose, even beyond 200 *μ*mol/L; however, at all treatment doses, the number of cells recorded were lower than that recorded in untreated MSC/PLA (Figures 5(a) and 5(b)). Based on these findings, 200 *μ*mol/L of VC was chosen as the optimal dose for evaluating the suppressive effect on cell senescence.

We evaluated the protein level of prelamin A in SCB-MSCs cell senescence after VC treatment. Interestingly, VC dramatically alleviated the expression of prelamin A in MSC/PLA (Figure 5(c)). To determine the effect of VC on aging-related parameters, we pretreated MSC/PLA with VC and examined different cellular properties. Consistent with the changes observed in premature cellular senescence, VC treatment substantially alleviated SASP, including the mRNA levels of pro-inflammatory cytokines such as TNF*α* and IL1*β* (Figure 5(d)), increased the expression of Ki67 (Figure 5(e)), reversed the expression of POT1, RAP1A, and TERF1 in mRNA level, implying the rescue protection of shelterin complex to telosome (Figure 5(f)). VC also significantly elevated collagen I and collagen III mRNA levels, reconstructing ECM defect induced by prelamin A overexpression (Figure 5(g)).

Association with cell cycle-related genes suggested VC treatment downregulated p21 protein level (Figure 5(h)), and upregulated CDK2 and CCNE1 mRNA levels (Figure 5(i)). VC also changed the expression of Bcl2, and BAX at the mRNA levels, with decreased levels of the apoptotic Bcl2/BAX ratio (Figure 5(j)), thereby slightly rescuing the change induced by prelamin A overexpression. But, VC had no effect on GADD45A expression (data not shown), suggesting that the restoration of nuclear lamina components may not be associated with alleviation of DNA damage response (DDR).

To investigate whether VC can restore the neovascularization of SCB-MSCs *in vivo*, VC-pretreated MSC/PLA (MSC/PLA+VC) were transplanted in hind-limb ischemia, *n* = 7 per group. At day 7, ischemic hind limbs in the MSC/PLA+VC group showed increased limb skin temperature and swelling, compared to the MSC/PLA group, even higher blood prefusion than that in the normal limb. But no significant increase in blood perfusion was observed on day 14 compared to the MSC/PLA group, which was still inferior to the MSC/GFP group, even at day 21 (Figures 5(k) and 5(l)), suggesting serious functional defects in MSC/PLA *in vivo*.

**Figure 6:**
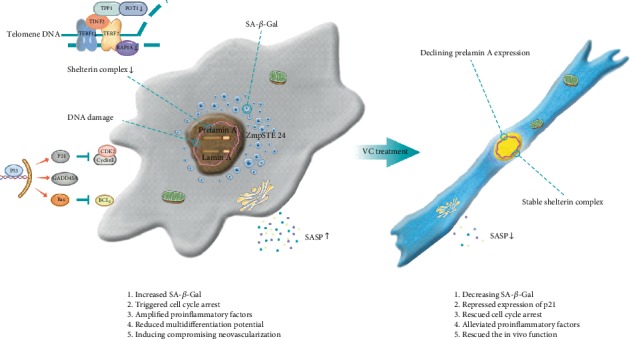
A proposed model illustrates the aging properties in SCB-MSCs induced by prelamin A overexpression and partly rescued by VC treatment.

Together, our findings showed that prelamin A triggered SCB-MSC premature senescence and VC inhibited prelamin A expression in MSC/PLA, which retarded the increased cellular aging. VC treatment partly decreased SASP in MSC/PLA, reversed the decreased expression of shelterin complex, and partly rescued cell cycle arrest and resistance to apoptosis (Figure 6).

## 5. Discussion

Aging is a predominant risk factor for many chronic conditions. Stem cell senescence plays a pivotal role in the aging process. In the present study, we found that VC treatment could rescue prelamin A-induced premature senescence of SCB-MSCs. SCB-MSCs with accumulated prelamin A displayed reduced multidifferentiation potential and compromised neovascularization in hind-limb ischemia. Overexpression of prelamin A induced abnormal nuclear morphology, a decrease in expression of the shelterin complex, accelerated DNA damage, and cell cycle arrest leading to SCB-MSCs senescence. Interestingly, VC slightly improved the neovascularization of MSC/PLA in hind-limb ischemia. VC alleviated senescence by sharply decreasing the expression of prelamin A, decreasing SASP, and rescuing the damaging effect on shelterin complex and cell cycle arrest induced by prelamin A overexpression. Though, VC has been widely used in protecting cells from oxidation, our study, for the first time, identified that VC can be a gene-protective agent against prelamin A accumulation.

### 5.1. Prelamin A Overexpression Is a Potent Driver of SCB-MSC Cell Senescence and Function Exhaust

In Hutchinson–Gilford progeria syndrome (HGPS) and normal aging, VSMCs, and fibroblasts, premature senescence can be attributed to the toxic accumulation of prelamin A and progerin [15, 40]. Similar to this study, in early-passage (p3) of SCB-MSCs, little prelamin A were detected, but it mounted rapidly in later-passage (p6), followed by limited growth characteristics and short lifespan. Increased expression of SA-*β*-Gal activity, an aging marker in later-passages, verified cell senescence. The inferior proliferation of MSCs isolated from adult tissue is representative of the characters of adult stem cell after aging [41], and MSCs isolated from abandoned tibial plateau of patients undergoing knee replacement surgery (aged from 50-60 years) were chosen in this study.

As expected, prelamin A occurred concomitantly with an increase in premature senescence. Nuclear dysmorphisms, including nuclear lobulation, nuclear enlargement, and morphology defects, were detected in prelamin A overexpressed SCB-MSCs, thus being consistent with the changes in VSMCs, human umbilical vein endothelial cells (HUVEC), and endothelial colony-forming cells (ECFC) as reported by previous studies [15, 29, 42]. Prelamin A overexpression had no effect in the basic expression of stem cell markers, including CD73, CD90, CD105, and CD44. Melanoma cell-adhesion molecule (MCAM, also known as CD146) is the marker of antiaging and stemness and has been shown to gradually decrease after multiple passages of MSCs cultured from human umbilical cord blood [43]. However, very low positive expression was observed in SCB-MSCs, leading to no difference between the two groups (data not shown), indicating that SCB-MSCs obtained from donors undergoing knee replacement surgery were prone to senescence.

Key features of cell senescence, including decline in proliferation and cell cycle arrest, were observed in SCB-MSCs accumulated prelamin A. Previous studies revealed that prelamin A induced the decrease in expression of Ki67 in VSMCs [29] and BrdU in HUVEC and ECFC [42]. Similarly, our study showed reduced expression of Ki67 in SCB-MSCs-accumulated prelamin A, suggesting reduction of proliferative capacity.

Extensive studies have shown that cells with persistent DNA damage develop a specific SASP. *In vitro* studies using various cell types indicate that an accumulation of senescent cells with critically short telomere may produce proinflammatory factors, which in turn could contribute to an increased inflammatory load [44–47]. In our study, dramatic increase of IL6, IL8, and IL1*β* demonstrated the SASP and accelerated cell senescence.

Previous reports showed that donor age did not affect the ability of the cells to form bone *in vivo* [41] and in early-passage cultures of MSC obtained from young and old donors treated with osteogenic differentiation medium [48]. However, in iPS-MSC lines, osteogenic differentiation varied [17]. Long-term treatments with some HIV protease inhibitors induced senescence and altered osteoblast and adipocyte differentiation [49]. In our study, the osteogenic and adipogenic potential in prelamin A-accumulated SCB-MSCs decreased significantly after being cultured in differentiation medium, suggesting lower multidifferentiation potential after prelamin A accumulation. This partly agrees with previous studies showing that progerin interfered with the adipogenic potential of human MSCs [50, 51]. The difference in these studies could be partly due to the different sources of MSCs and the prelamin A accumulation.

Using a hind-limb ligation mouse model, we found that the MSC/PLA group was only slightly better in rescuing the hind-limb ischemia than the PBS group, in contrast to the successful rescue mediated by the MSC/GFP group. Though more limbs salvage were seen in SCB-MSCs groups, the MSC/PLA group showed more foot necrosis than the MSC/GFP group. Blood perfusion assayed by Laser-Doppler flow images showed the inferior rescue by the MSC/PLA group. Histological analysis, using hematoxylin, eosin, and Masson trichrome staining, verified infiltration of numerous granulocytes and neutrophils fibrosis, and muscle degeneration decreased in SCB-MSCs compared with the PBS group. Similarly, inferior rescue was observed in the MSC/PLA group compared to that in the MSC/GFP group. Immunostaining of CD31 revealed overall neovascularization that was not significantly different between the MSC/GFP group and the MSC/PLA group, which was superior to PBS group, and the delayed and impaired reconstruction of microcirculation may be responsible for it.

### 5.2. Prelamin A-Induced Amplification of the DNA Damage and Shelterin Complex Damage Accelerated SCB-MSC Cell Cycle Arrest

Previous studies show that prelamin A accumulated in different cells induced abnormal nuclear morphology and triggered DNA damage [16, 19, 29, 42]. Chromatin surrounding the double-strand breaks (DSBs) is altered and histones are modified to facilitate access for repair proteins, after DNA damage [52]. As a rapid response to DSB induction, H2AX is phosphorylated at Ser139 forming *γ*-H2AX to facilitate the recruitment of DNA repair proteins, such as 53BP1 and BRCA1 [30]. More *γ*-H2AX and 53BP1 were detected in prelamin A-overexpressed SCB-MSCs, suggesting higher amplified DNA damage.

Telomere dysfunction is one of the key hallmarks during aging and is induced by either critical shortening or disruption of the shelterin complex, leading to cellular senescence [53]. Mammalian telomeres are associated with Shelterin, a protein complex that functions to protect DNA ends from being recognized as double-strand breaks that would trigger a DNA damage response [32, 33]. Previous studies revealed that loss of POT1 function during G1 leads to rapid telomere erosion during the ensuing S/G2 period [34]. The levels of three of the six components in telomere attrition associated shelterin complex, POT1, RAP1A, and TERF1 decreased, indirectly suggesting telomere dysfuction.

DNA damage (single or double-strand breaks) triggers adapted cellular responses, triggering cell cycle arrest, senescence, apoptosis, or proliferation. These responses are elicited through different signaling pathways, activating cell cycle checkpoints, subsequently leading to different cellular fates: cell cycle arrest promoting senescence (permanent arrest) or cell death. Interestingly, apoptosis was inhibited in MSC/PLA, as shown by flow cytometry, following increased levels of the apoptotic Bcl2/BAX ratio and decreased expression of caspase 3. S phase arrest was detected in SCB-MSCs overexpressing prelamin A. Senescent cells are characterized by a persistent DNA damage response (DDR), which leads to eventual cell cycle arrest and senescence through activation of the p53/p21 and p16/pRb pathways [54]. Upregulation of the cyclin-dependent kinase inhibitor p21 and p53 contributed to cell cycle arrest and decreased proliferation, supporting the hypothesis that these alterations, including chronic activation of the p53 pathway, may be the drivers of the senescent phenotype. As expected, CDK2 and CCNE decreased sharply, suggesting that the G1/S checkpoint was more sensitive to prelamin A overexpression. It has been reported that prelamin A accumulation in VSMC induces mitotic failure [29]. However, our observations in SCB-MSCs that G1/S phase was retarded, considering different cells, need further studies.

Microarray analysis reveals changes in gene expression profile, including alterations in DNA replication, cell cycle, and amplified inflammatory factors in prelamin A-overexpressed SCB-MSCs, verified above.

### 5.3. VC Treatment Partly Rescue Prelamin A-Induced Compromised Function of SCB- MSCs

High levels of VC tend to induce free radicals, a phenotypic switch from antioxidant to oxidizing agent. Vitamin C (ranged from 0.1 *μ*M to 10 mM) was used in cultured cell for the therapy of cancer cells. In our study, we tried different doses of Vitamin C and found that 200 *μ*M of VC was the optimal dose for evaluating the suppressive effect on cell senescence. According to the content of ascorbate (VC) in human tissues, 200 *μ*M concentration approximately equals with the concentration of VC in the cerebral spinal fluid, which is 40-80 *μ*M in plasma [55]. Our study shows that high concentrations of VC (i.e., 200 *μ*mol/L) produce the best effect on rejuvenating SCB-MSCs without discernible cytotoxicity, which means that oral supplement of VC might not be enough to retard cell senescence. Thus, the titration for optimal VC concentration in human patients needs to be done very cautiously.

We identified a high dose of VC as a potent agent to alleviate aging process in a stem cell model for adult aging. VC has the significant effect on repressing cellular senescence (indicated by SA-*β*-Gal staining), on decreasing the expression of prelamin A, on decreasing SASP, and on rescuing the damage in shelterin complex induced by prelamin A overexpression.

We also identified that VC decreased the TNF*α*, IL-8, and IL1*β* secretion in MSC/PLA, which maybe contributed to the improvement of MSC/PLA in treating hind-limb ischemia. This suggests a possibility of using VC to decrease the inflammatory mediators in the seniors. Given a widely reported fact that MSCs support a significant paracrine effect through the secretion of proangiogenic cytokines which provide antiapoptotic effects and stimulate revascularization [56], possibly, VC slightly restores the in vivo viability of MSC/PLA might though reduced inflammatory mediators expression induced by PLA overexpression.

In summary, we have shown that prelamin A accumulation in SCB-MSCs lead to abnormal nuclear morphology and inferior multipotential differentiation of MSCs both *in vitro* and *in vivo* and injuring the stabilization of shelterin complex, accelerating the DNA damage, and invoking cell cycle arrest. Intriguingly, parts of these changes were rescued by VC treatment. As prelamin A accumulation existed widely both in premature aging and physiological human aging, VC plays different roles when used in low and high doses. Further studies are required to understand senescent phenotypes and aging-related pathologies to develop therapeutic strategies. What is more, how to supply enough VC effectively to antisenescence and how VC coordinates different mechanisms in alleviating aging defects in MSCs also require further investigation.

## Data Availability

The data used to support the findings of this study are available from the corresponding author upon request.

## Supplementary Materials

Supplementary Materials Figure S1. SCB-MSCs characteristics and prelamin A transfection efficiency. (a) Morphology of primary SCB-MSCs. Scale bar: 500. (b) Immunophenotypic features of mesenchymal markers in SCB-MSCs. (c) Limited proliferation of SCB-MSCs after in vitro subculturing. (d) Alizarin red staining (up, scale bar: 500 *μ*m) and oil red staining (down, scale bar: 100 *μ*m) display the osteogenic and adipogenic differentiation of SCB-MSCs, respectively. (e) Visualization of transfection efficiency by immunofluorescence and flow cytometry. Scale bar: 200 *μ*m. (f) Immunoblots confirmed the overexpression of prelamin A in SCB-SMCs. Table S1: basic information of the donors. Table S2: primers' list. Table S3: significant changes in the levels of 386 mRNA (193 upregulated, 193 downregulated).

## References

[1] Kennedy B. K., Berger S. L., Brunet A. (2014). Geroscience: linking aging to chronic disease. *Cell*.

[2] Oh J., Lee Y. D., Wagers A. J. (2014). Stem cell aging: mechanisms, regulators and therapeutic opportunities. *Nature Medicine*.

[3] López-Otín C., Blasco M. A., Partridge L., Serrano M., Kroemer G. (2013). The hallmarks of aging. *Cell*.

[4] Farr J. N., Xu M., Weivoda M. M. (2017). Targeting cellular senescence prevents age-related bone loss in mice. *Nature Medicine*.

[5] Roos C. M., Zhang B., Palmer A. K. (2016). Chronic senolytic treatment alleviates established vasomotor dysfunction in aged or atherosclerotic mice. *Aging Cell*.

[6] Childs B. G., Baker D. J., Wijshake T., Conover C. A., Campisi J., van Deursen J. (2016). Senescent intimal foam cells are deleterious at all stages of atherosclerosis. *Science*.

[7] Chang J., Wang Y., Shao L. (2016). Clearance of senescent cells by ABT263 rejuvenates aged hematopoietic stem cells in mice. *Nature Medicine*.

[8] Jeon O. H., Kim C., Laberge R. M. (2017). Local clearance of senescent cells attenuates the development of post-traumatic osteoarthritis and creates a pro-regenerative environment. *Nature Medicine*.

[9] Bustos M. L., Huleihel L., Kapetanaki M. G. (2014). Aging mesenchymal stem cells fail to protect because of impaired migration and antiinflammatory response. *American Journal of Respiratory and Critical Care Medicine*.

[10] Stolzing A., Jones E., McGonagle D., Scutt A. (2008). Age-related changes in human bone marrow-derived mesenchymal stem cells: consequences for cell therapies. *Mechanisms of Ageing and Development*.

[11] Schreiber K. H., Kennedy B. K. (2013). When lamins go bad: nuclear structure and disease. *Cell*.

[12] Broers J. L., Ramaekers F. C. S., Bonne G., Yaou R. B., Hutchison C. J. (2006). Nuclear lamins: laminopathies and their role in premature ageing. *Physiological Reviews*.

[13] Kubben N., Misteli T. (2017). Shared molecular and cellular mechanisms of premature ageing and ageing-associated diseases. *Nature Reviews Molecular Cell Biology*.

[14] Auclair M., Afonso P., Capel E., Caron-Debarle M., Capeau J. (2014). Impact of darunavir, atazanavir and lopinavir boosted with ritonavir on cultured human endothelial cells: beneficial effect of pravastatin. *Antiviral Therapy*.

[15] Liu Y., Drozdov I., Shroff R., Beltran L. E., Shanahan C. M. (2013). Prelamin A accelerates vascular calcification via activation of the DNA damage response and senescence-associated secretory phenotype in vascular smooth muscle cells. *Circulation Research*.

[16] Osorio F. G., Bárcena C., Soria-Valles C. (2012). Nuclear lamina defects cause ATM-dependent NF-*κ*B activation and link accelerated aging to a systemic inflammatory response. *Genes & Development*.

[17] Zhang J., Lian Q., Zhu G. (2011). A human iPSC model of Hutchinson Gilford Progeria reveals vascular smooth muscle and mesenchymal stem cell defects. *Cell Stem Cell*.

[18] Lee J. Y., Yu K. R., Lee B. C. (2018). GATA4-dependent regulation of the secretory phenotype via MCP-1 underlies lamin A-mediated human mesenchymal stem cell aging. *Experimental & Molecular Medicine*.

[19] Infante A., Gago A., de Eguino G. R. (2014). Prelamin A accumulation and stress conditions induce impaired Oct-1 activity and autophagy in prematurely aged human mesenchymal stem cell. *Aging*.

[20] Corre I., Paris F., Huot J. (2017). The p38 pathway, a major pleiotropic cascade that transduces stress and metastatic signals in endothelial cells. *Oncotarget*.

[21] Zhu H., Guo Z. K., Jiang X. X. (2010). A protocol for isolation and culture of mesenchymal stem cells from mouse compact bone. *Nature Protocols*.

[22] Ramirez-Zacarias J. L., Castro-Munozledo F., Kuri-Harcuch W. (1992). Quantitation of adipose conversion and triglycerides by staining intracytoplasmic lipids with Oil red O. *Histochemistry*.

[23] Kim Y., Kim H., Cho H., Bae Y., Suh K., Jung J. (2007). Direct comparison of human mesenchymal stem cells derived from adipose tissues and bone marrow in mediating neovascularization in response to vascular ischemia. *Cellular Physiology and Biochemistry*.

[24] Dominici M., le Blanc K., Mueller I. (2006). Minimal criteria for defining multipotent mesenchymal stromal cells. The International Society for Cellular Therapy position statement. *Cytotherapy*.

[25] Freund A., Orjalo A. V., Desprez P. Y., Campisi J. (2010). Inflammatory networks during cellular senescence: causes and consequences. *Trends in Molecular Medicine*.

[26] Waterman R. S., Henkle S. L., Betancourt A. M. (2012). Mesenchymal stem cell 1 (*MSC1*)-based therapy attenuates tumor growth whereas *MSC2*-treatment promotes tumor growth and metastasis. *PLoS One*.

[27] Lian Q., Zhang Y., Zhang J. (2010). Functional mesenchymal stem cells derived from human induced pluripotent stem cells attenuate limb ischemia in mice. *Circulation*.

[28] Limbourg A., Korff T., Napp L. C., Schaper W., Drexler H., Limbourg F. P. (2009). Evaluation of postnatal arteriogenesis and angiogenesis in a mouse model of hind-limb ischemia. *Nature Protocols*.

[29] Ragnauth C. D., Warren D. T., Liu Y. (2010). Prelamin A acts to accelerate smooth muscle cell senescence and is a novel biomarker of human vascular aging. *Circulation*.

[30] Celeste A., Petersen S., Romanienko P. J. (2002). Genomic instability in mice lacking histone H2AX. *Science*.

[31] Krishnan V., Chow M. Z., Wang Z. (2011). Histone H4 lysine 16 hypoacetylation is associated with defective DNA repair and premature senescence in Zmpste24-deficient mice. *Proceedings of the National Academy of Sciences of the United States of America*.

[32] Diotti R., Loayza D. (2011). Shelterin complex and associated factors at human telomeres. *Nucleus*.

[33] de Lange T. (2005). Shelterin: the protein complex that shapes and safeguards human telomeres. *Genes & Development*.

[34] Pitt C. W., Cooper J. P. (2010). Pot1 inactivation leads to rampant telomere resection and loss in one cell cycle. *Nucleic Acids Research*.

[35] Wang C., Jurk D., Maddick M., Nelson G., Martin-Ruiz C., von Zglinicki T. (2009). DNA damage response and cellular senescence in tissues of aging mice. *Aging Cell*.

[36] Albrechtsen N., Dornreiter I., Grosse F., Kim E., Wiesmüller L., Deppert W. (1999). Maintenance of genomic integrity by p53: complementary roles for activated and non-activated p53. *Oncogene*.

[37] Li Y., Zhang W., Chang L. (2016). Vitamin C alleviates aging defects in a stem cell model for Werner syndrome. *Protein & Cell*.

[38] Dallaire A., Proulx S., Simard M. J., Lebel M. (2014). Expression profile of Caenorhabditis elegans mutant for the Werner syndrome gene ortholog reveals the impact of vitamin C on development to increase life span. *BMC Genomics*.

[39] Massip L., Garand C., Paquet E. R. (2010). Vitamin C restores healthy aging in a mouse model for Werner syndrome. *The FASEB Journal*.

[40] Scaffidi P., Misteli T. (2006). Lamin A-dependent nuclear defects in human aging. *Science*.

[41] Stenderup K., Justesen J., Clausen C., Kassem M. (2003). Aging is associated with decreased maximal life span and accelerated senescence of bone marrow stromal cells. *Bone*.

[42] Bonello-Palot N., Simoncini S., Robert S. (2014). Prelamin A accumulation in endothelial cells induces premature senescence and functional impairment. *Atherosclerosis*.

[43] Jin H. J., Kwon J. H., Kim M. (2016). Downregulation of melanoma cell adhesion molecule (MCAM/CD146) accelerates cellular senescence in human umbilical cord blood-derived mesenchymal stem cells. *Stem Cells Translational Medicine*.

[44] Childs B. G., Gluscevic M., Baker D. J. (2017). Senescent cells: an emerging target for diseases of ageing. *Nature Reviews Drug Discovery*.

[45] van Deursen J. M. (2014). The role of senescent cells in ageing. *Nature*.

[46] Tchkonia T., Zhu Y., van Deursen J., Campisi J., Kirkland J. L. (2013). Cellular senescence and the senescent secretory phenotype: therapeutic opportunities. *The Journal of Clinical Investigation*.

[47] Krtolica A., Parrinello S., Lockett S., Desprez P. Y., Campisi J. (2001). Senescent fibroblasts promote epithelial cell growth and tumorigenesis: a link between cancer and aging. *Proceedings of the National Academy of Sciences of the United States of America*.

[48] Justesen J., Stenderup K., Eriksen E. F., Kassem M. (2002). Maintenance of osteoblastic and adipocytic differentiation potential with age and osteoporosis in human marrow stromal cell cultures. *Calcified Tissue International*.

[49] Hernandez-Vallejo S. J., Beaupere C., Larghero J., Capeau J., Lagathu C. (2013). HIV protease inhibitors induce senescence and alter osteoblastic potential of human bone marrow mesenchymal stem cells: beneficial effect of pravastatin. *Aging Cell*.

[50] Scaffidi P., Misteli T. (2008). Lamin A-dependent misregulation of adult stem cells associated with accelerated ageing. *Nature Cell Biology*.

[51] de Eguino G. R., Infante A., Schlangen K. (2012). Sp1 transcription factor interaction with accumulated prelamin a impairs adipose lineage differentiation in human mesenchymal stem cells: essential role of sp1 in the integrity of lipid vesicles. *Stem Cells Translational Medicine*.

[52] Downs J. A., Nussenzweig M. C., Nussenzweig A. (2007). Chromatin dynamics and the preservation of genetic information. *Nature*.

[53] Jones M., Bisht K., Savage S. A., Nandakumar J., Keegan C. E., Maillard I. (2016). The shelterin complex and hematopoiesis. *The Journal of Clinical Investigation*.

[54] Rodier F., Coppé J. P., Patil C. K. (2009). Persistent DNA damage signalling triggers senescence-associated inflammatory cytokine secretion. *Nature Cell Biology*.

[55] Du J., Cullen J. J., Buettner G. R. (2012). Ascorbic acid: chemistry, biology and the treatment of cancer. *Biochimica et Biophysica Acta*.

[56] Al-Rifai R., Nguyen P., Bouland N. (2019). In vivo efficacy of endothelial growth medium stimulated mesenchymal stem cells derived from patients with critical limb ischemia. *Journal of Translational Medicine*.

