# Supplementary material for: Vitamin C Treatment Rescues Prelamin A-Induced Premature Senescence of Subchondral Bone Mesenchymal Stem Cells
Source: Stem Cells Int. 2020 Apr 3;2020:3150716. doi: 10.1155/2020/3150716 (PMC7157810; doi:10.1155/2020/3150716)
Supplement: Supplementary Materials — Figure S1. SCB-MSCs characteristics and prelamin A transfection efficiency. (a) Morphology of primary SCB-MSCs. Scale bar: 500. (b) Immunophenotypic features of mesenchymal markers in SCB-MSCs. (c) Limited proliferation of SCB-MSCs after in vitro subculturing. (d) Alizarin red staining (up, scale bar: 500 μm) and oil red staining (down, scale bar: 100 μm) display the osteogenic and adipogenic differentiation of SCB-MSCs, respectively. (e) Visualization of transfection efficiency by immunofluorescence and flow cytometry. Scale bar: 200 μm. (f) Immunoblots confirmed the overexpression of prelamin A in SCB-SMCs. Table S1: basic information of the donors. Table S2: primers' list. Table S3: significant changes in the levels of 386 mRNA (193 upregulated, 193 downregulated). [file 3150716.f1.pdf]

Figure S1

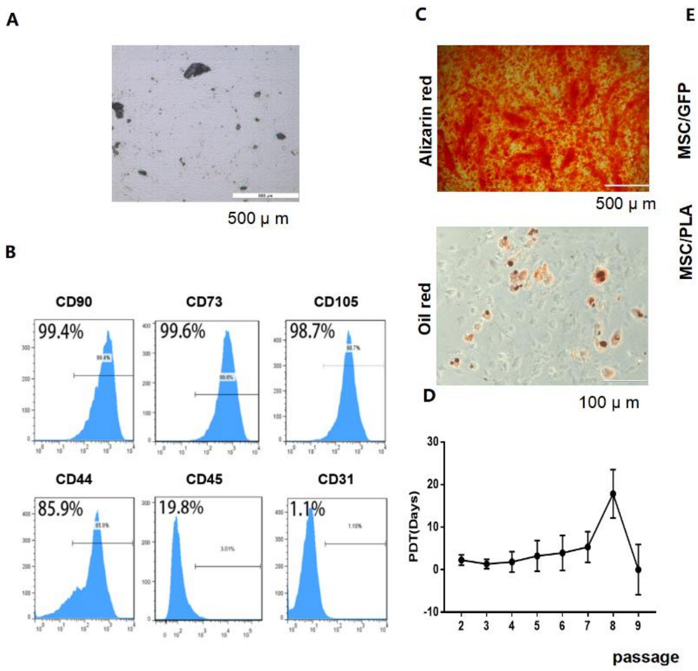

A
500 μ m
B
CD90
99.4%
CD73
99.6%
CD105
98.7%
CD44
85.9%
CD45
19.8%
CD31
1.1%
C
Alizarin red
500 μ m
Oil red
100 μ m
D
PDT(Days)
passage
E
MSC/GFP
57.8%
MSC/PLA
87.5%
200 μ m
F
MSC/GFP
MSC/PLA
Prelamin A
Lamin A/C
β-actin

Table S1

Basic information of the donors

Years	gender					
55	male					
60	male					
52	femal					
57	male					
55	femal					

Table S2

Primers List

Oligo Name	Sequence(5' to 3')
F-OCN	cag gcg cta cct gta tca atg gc
R-OCN	gcc gat gtg gtc agc caa ctc
F-RUNX2	aac agc agc agc agc agc ag
R-RUNX2	gca ccg agc aca gga agt tgg
F-OPN	agc gag gag ttg aat ggt gca tac
R-OPN	aat ctg gac tgc ttg tgg ctg tg
Hcollagen III-F	tgc tgc tgg tac tcc tgg tct g
Hcollagen III-R	acc tgg acc gcc tgg ttc ac
Hcollagen I-F	aaa gat gga ctc aac ggt ctc
Hcollagen I-R	cat cgt gag cct tct ctt gag
H-BCL2 -F	gac ttc gcc gag atg tcc ag
H-BCL2-R	gaa ctc aaa gaa ggc cac aat c
H-CASP3-F	cca aag atc ata cat gga agc g
H-CASP3-R	ctg aat gtt tcc ctg agg ttt g
H-BAX-F	cga act gga cag taa cat gga g
H-BAX-R	cag ttt gct ggc aaa gta gaa a
H-P21-F	gat gga act tcg act ttg tca c
H-P21-R	gtc cac atg gtc ttc ctc tg
H-GADD45A-F	cag atc cac ttc acc ctg atc
H-GADD45A-R	gat gaa tgt gga ttc gtc acc a
HP16-F	ggc cga tcc agg tca tga tga tg
HP16-R	cac cag cgt gtc cag gaa gc
Hp-53-F	cctcagcatcttatccgagtgg
Hp-53-R	tggatggtggtacagtcagagc

H-CCND1-F	tct aca ccg aca act cca tcc g
H-CCND1-R	tct ggc att ttg gag agg aag tg
H-CCNE-F	tgt gtc ctg gat gtt gac tgc c
H-CCNE1-R	ctc tat gtc gca cca ctg ata cc
H-CDK2-F	atg gat gcc tct gct ctc act g
H-CDK2-R	ccc gat gag aat ggc aga aag c
H-CDK4-F	cca tca gca cag ttc gtg agg t
H-CDK4-R	tca gtt cgg gat gtg gca cag a
H-CDK6-F	gga taa agt tcc aga gcc tgg ag
H-CDK6-R	gcg atg cac tac tcg gtg tga a
H-IL6-F	aga cag cca ctc acc tct tca g
H-IL6-R	ttc tgc cag tgc ctc ttt gct g
H-IL8-F	gag agt gat tga gag tgg acc ac
H-IL8-R	cac aac cct ctg cac cca gtt t
H-TERF2-F	gtg gaa aag cca ccc aga gaa c
H-TERF2-R	tgc aaa ggc tgc ctc aga atc c
H-TERF1-F	cat gga acc cag caa caa gac c
H-TERF1--R	ctg ctt tca gtg gct ctt ctg c
H-POT1-F	cag aac ctg acg aca gct ttc c
H-POT1-R	gca cat agt ggt gtc ctc tcc a
H-RAP1A-F	act tac agg acc tga ggg aac ag
H-RAP1A-R	cct gct ctt tgc caa cta ctc g
H-ARID1A -F	aag cca cca act cca gca tcc a
H-ARID1A -R	cgc ttc tgg aat gtg gag tca c
H-TINF2-F	gtcagaggctcctgtggatttg
H-TINF2-R	gtgtaggcagtgctttctccag
H-TPP1-F	ggtggcttcagcaatgtgttcc
H-TPP1-R	gaagtaactggatggtggcagg
H-PPAR-γ-F	agcctgcgaaagccttttggtg
H-PPAR-γ-R	ggcttcacattcagcaaacctgg
H-CEBPA-F	aggaggatgaagccaagcagct
H-CEBPA-R	agtgcgcgatctggaactgcag

Table S3

Significant changes in the levels of 386 mRNA (193 upregulated, 193 downregulated)

ProbeID	GeneSymbol	Regulation(PLA_vs._CTRL)
hsa_bmr020401	AAMDC	up
hsa_gmr049805	ABCA2	up
hsa_gmr043452	ABCC11	up
hsa_gmr016953	ABCC8	down
hsa_gmr065377	ABCC9	down
hsa_gmr038136	ACSL6	up
hsa_gmr041256	ADAM12	down
hsa_gmr024054	ADAMTS12	down
hsa_gmr088550	ADAMTS14	down
hsa_gmr088553	ADAMTS14	down
hsa_gmr088554	ADAMTS14	down
hsa_gmr088555	ADAMTS14	down
hsa_gmr088556	ADAMTS14	down
hsa_gmr088552	ADAMTS14	down
hsa_gmi128679	ADAMTS14	down
hsa_gmr044494	ADAMTS14	down
hsa_gmr088551	ADAMTS14	down
hsa_gmr097776	ADGRL3	down
hsa_gmr079573	AGBL2	up
hsa_gmr021258	AIF1	down
hsa_gmr092496	ALPL	down
hsa_bmr009465	ALPL	down
hsa_gmr000424	ALPL	down
hsa_gmr037728	ARHGAP11A	up
hsa_gmr029105	ARMC9	down
hsa_gmr075586	ARMC9	down
hsa_gmr075585	ARMC9	down
hsa_gmr110205	ASPSCR1	up
hsa_gmr081860	ATAD5	up
hsa_gmr047161	ATP2A1	down
hsa_gmr076350	ATP8A1	down
hsa_gmr110973	B4GALT6	up
hsa_gmi127643	BAALC	down
hsa_gmr004203	BANK1	down
hsa_gmr098060	BANK1	down
hsa_gmr005390	BANK1	down
hsa_gmr014063	BCL2L13	up
hsa_bmr000571	BDKRB2	up

hsa_gmr103644	BEND7	up
hsa_gmr008761	BIRC5	up
hsa_gmr007757	BNIPL	down
hsa_gmr092670	BRINP3	down
hsa_gmr105453	BRSK2	down
hsa_gmr104579	C11orf65	down
hsa_gmr086397	C13orf46	down
hsa_gmr106747	C2CD5	down
hsa_bmr049840	C2orf66	down
hsa_gmr075869	C3orf58	down
hsa_gmr099003	C5orf63	down
hsa_gmr004611	C7orf57	down
hsa_gmi050523	CABLES1	up
hsa_gmr047054	CAMKK2	up
hsa_bmr116289	CARD17	down
hsa_gmr025644	CBX7	up
hsa_gmr064294	CCDC125	up
hsa_gmr090360	CCDC125	up
hsa_gmr121578	CCDC125	up
hsa_gmr119981	CCDC138	down
hsa_gmr102607	CCDC171	up
hsa_gmr040302	CCHCR1	up
hsa_gmr018058	CCL4L2	down
hsa_gmr017750	CD36	down
hsa_gmi130155	CDCA3	up
hsa_gmi136247	CDCA3	up
hsa_gmi130156	CDCA3	up
hsa_gmi136248	CDCA3	up
hsa_gmi136249	CDCA3	up
hsa_gmr020534	CDH10	down
hsa_gmr029837	CELF4	up
hsa_gmr090503	CENPK	up
hsa_gmr041515	CENPK	up
hsa_gmr099166	CENPK	up
hsa_gmr099165	CENPK	up
hsa_gmr011648	CIT	up
hsa_gmr087839	CIT	up
hsa_gmi059451	CLEC12B	down
hsa_gmr029589	CLEC2L	down
hsa_bmr044171	CLNK	up
hsa_gmr017171	COL4A6	down
hsa_gmr066749	COMMD7	up
hsa_gmr042630	CPEB1	down
hsa_gmr003812	CPEB1	down

hsa_bmr115625	CREM	down
hsa_gmr004362	CRTC1	up
hsa_bmr047051	CSF1	up
hsa_gmr000679	CSF1	up
hsa_gmr047049	CSF1	up
hsa_gmr091400	CTSF	up
hsa_gmr033172	CTSF	up
hsa_gmr002966	CXCL12	up
hsa_gmr009618	CXCL12	up
hsa_bmr049056	CXCL12	up
hsa_gmi051784	DCAF11	up
hsa_gmr075645	DGKD	up
hsa_gmr095689	DGKD	up
hsa_gmr075649	DGKD	up
hsa_gmr046512	DGKD	up
hsa_gmr033045	DGKD	up
hsa_gmr063623	DGKD	up
hsa_gmr104604	DLG2	down
hsa_gmr020505	DLK1	down
hsa_gmr005237	DMKN	up
hsa_gmr011688	DNMT3B	down
hsa_gmr068006	DOK3	down
hsa_gmr099317	DOK3	down
hsa_gmr031426	DSG2	down
hsa_gmr002672	DUT	up
hsa_gmr005782	DYSF	down
hsa_gmr005899	DYSF	down
hsa_gmr067672	DYSF	down
hsa_gmr067673	DYSF	down
hsa_gmr005901	DYSF	down
hsa_gmr005902	DYSF	down
hsa_gmr005903	DYSF	down
hsa_gmr005904	DYSF	down
hsa_gmr005906	DYSF	down
hsa_gmr005907	DYSF	down
hsa_gmr005905	DYSF	down
hsa_gmr005910	DYSF	down
hsa_gmr032925	DYSF	down
hsa_gmr005908	DYSF	down
hsa_bmr030952	EFNB3	up
hsa_gmr079424	EHF	down
hsa_gmr105088	ELMOD1	down
hsa_gmr071315	ELOVL7	down
hsa_gmr014113	EPGN	down

hsa_gmr014114	EPGN	down
hsa_gmr014115	EPGN	down
hsa_gmr014117	EPGN	down
hsa_gmi054886	EPGN	down
hsa_gmi054888	EPGN	down
hsa_gmr120613	EPHB1	down
hsa_gmr104060	FAM196A	down
hsa_gmr046166	FAM213B	up
hsa_gmr080794	FANCA	up
hsa_gmr104524	FAT3	down
hsa_gmr043314	FAXDC2	up
hsa_gmr071331	FAXDC2	up
hsa_gmr086372	FBRSL1	up
hsa_gmr086370	FBRSL1	up
hsa_gmr086366	FBRSL1	up
hsa_gmi058504	FBXO44	up
hsa_gmr069729	FLRT1	down
hsa_gmr010297	FLVCR2	down
hsa_bmr034677	FRAT1	up
hsa_gmr013141	GDAP1L1	down
hsa_gmr117056	GPR18	down
hsa_gmr031570	GPX3	down
hsa_gmr024401	GPX3	down
hsa_bmr105974	HCFC2	up
hsa_gmr006332	HDAC11	up
hsa_gmr093136	HHAT	down
hsa_gmr093130	HHAT	down
hsa_gmr093135	HHAT	down
hsa_gmr093131	HHAT	down
hsa_gmr008887	HHAT	down
hsa_bmr007914	HIST2H2BF	down
hsa_bmr000171	ICAM1	down
hsa_bmr001818	IGFBPL1	down
hsa_gmi137035	IGFL2	down
hsa_gmr075970	IGSF10	down
hsa_bmr000513	IL1A	down
hsa_gmr047163	IL37	up
hsa_gmr010718	INMT	up
hsa_gmi124888	INTU	down
hsa_gmr002548	IQCB1	down
hsa_gmr019298	ISG20	up
hsa_gmr031723	ITGA5	down
hsa_gmr116699	ITGA5	down
hsa_gmr085839	ITPR1	down

hsa_gmr076223	KALRN	up
hsa_gmr065503	KDM2B	up
hsa_gmr026319	KIDINS220	up
hsa_gmr026317	KIDINS220	up
hsa_bmi128866	LDB1	up
hsa_gmi130693	LINC00452	down
hsa_gmr095929	LOC105374811	down
hsa_gmi145870	LOC105374811	down
hsa_gmr095930	LOC105374811	down
hsa_bmr106765	LOC107984507	down
hsa_gmr014727	LRRC20	up
hsa_gmr086119	LRRFIP2	down
hsa_gmr070942	LRRFIP2	down
hsa_gmr070945	LRRFIP2	down
hsa_gmr070943	LRRFIP2	down
hsa_gmi059259	LRTOMT	up
hsa_gmr049344	MAP3K3	up
hsa_gmr031919	MAP3K3	up
hsa_gmr074443	MARC2	up
hsa_gmr025157	MARC2	up
hsa_bmr037113	METTL7A	up
hsa_gmr107330	MIPOL1	up
hsa_gmr107320	MIPOL1	up
hsa_gmr086788	MMS22L	up
hsa_bmr018924	MORN1	up
hsa_gmi054661	MS4A14	down
hsa_gmr074457	MSTO1	up
hsa_gmr024813	MTA3	up
hsa_gmr015928	MTA3	up
hsa_gmr024815	MTA3	up
hsa_gmr120256	MTA3	up
hsa_gmi141756	MTBP	up
hsa_gmr102357	MTSS1	up
hsa_gmr091225	MYO7A	down
hsa_gmr117148	N4BP2L1	up
hsa_gmr075215	NCKAP5	down
hsa_gmr010835	NCR2	down
hsa_gmr063470	NECTIN4	down
hsa_gmr074646	NECTIN4	down
hsa_gmr091964	NLRP3	up

hsa_gmr033860	NOTCH4	down
hsa_gmr037063	NRG2	up
hsa_gmr071109	NSD2	up
hsa_gmr072015	OBP2B	down
hsa_gmr002122	OLA1	up
hsa_gmr007244	OPRM1	down
hsa_bmr001516	OR2B3	down
hsa_bmr001456	OR52I1	down
hsa_bmr046751	OR5P2	down
hsa_gmr108722	OTOA	down
hsa_gmi056341	P4HA3	down
hsa_gmr017318	P4HA3	down
hsa_gmr119523	PCBP3	down
hsa_gmr015250	PCED1B	down
hsa_gmr068756	PCED1B	down
hsa_gmr106696	PCED1B	down
hsa_gmr111487	PDE4A	up
hsa_gmr035335	PDE4A	up
hsa_gmr006266	PIK3IP1	up
hsa_gmr044102	PIK3IP1	up
hsa_gmr118195	PIK3R6	down
hsa_gmr002007	PIK3R6	down
hsa_gmr082631	PINLYP	down
hsa_gmr116403	PKNOX2	down
hsa_gmr041434	PKNOX2	down
hsa_gmr116404	PKNOX2	down
hsa_gmr116405	PKNOX2	down
hsa_gmr116407	PKNOX2	down
hsa_gmr072495	PKNOX2	down
hsa_gmr090187	PKNOX2	down
hsa_gmr069291	PKNOX2	down
hsa_gmr105165	PKNOX2	down
hsa_gmr090186	PKNOX2	down
hsa_gmr090185	PKNOX2	down
hsa_gmr002731	PLEKHG1	down
hsa_gmr043570	PLXDC2	down
hsa_gmr014208	PPME1	down
hsa_gmi139335	PPME1	down
hsa_gmr105033	PPME1	down
hsa_gmi135872	PPP2R2D	up
hsa_gmr041485	PRDM16	up
hsa_gmr114737	PRKAR2B	up
hsa_gmi052996	PTPN7	up
hsa_gmr102978	PTPRD	down

hsa_gmr102967	PTPRD	down
hsa_gmr121031	PXYLP1	up
hsa_gmr046096	PXYLP1	up
hsa_gmr097443	PXYLP1	up
hsa_gmr002456	RAB28	up
hsa_gmr022978	RALGPS1	up
hsa_gmr093030	RALGPS2	up
hsa_gmr046388	RALGPS2	up
hsa_gmr016510	RALGPS2	up
hsa_gmr074446	RALGPS2	up
hsa_gmr070341	RALGPS2	up
hsa_gmr104447	RASGRP2	down
hsa_gmr047241	REM2	down
hsa_gmr089680	RNF220	down
hsa_gmr100305	ROS1	down
hsa_gmr095339	SCN2A	down
hsa_gmr084208	SEC14L6	down
hsa_gmr074565	SEMA4A	up
hsa_gmr033011	SEMA7A	down
hsa_gmr007516	SEMA7A	down
hsa_gmr007515	SEMA7A	down
hsa_gmr103009	SET	up
hsa_bmr014484	SETMAR	up
hsa_gmr067646	SFTPB	down
hsa_bmr007214	SHISA9	down
hsa_gmr065294	SIRT3	up
hsa_gmr080526	SLC24A1	up
hsa_gmr014247	SLC2A8	up
hsa_gmr000896	SLC2A9	up
hsa_bmr041905	SMIM2	down
hsa_gmr075121	SNED1	up
hsa_gmr110382	SPECC1	down
hsa_gmr076646	SPEF2	up
hsa_gmi124065	SSUH2	down
hsa_gmr067757	STAB1	down
hsa_gmi125521	STARD4	down
hsa_gmi058616	STARD4	down
hsa_gmr019909	STARD4	down
hsa_gmr019907	STARD4	down
hsa_gmr019906	STARD4	down
hsa_gmr019904	STARD4	down
hsa_gmi058615	STARD4	down
hsa_gmr121545	STARD4	down
hsa_gmr098638	STARD4	down

hsa_gmr066269	STAT5A	up
hsa_gmr098091	STK32B	down
hsa_gmr098092	STK32B	down
hsa_gmr039594	SUSD4	down
hsa_gmr072702	SYT1	down
hsa_gmr105457	SYT12	up
hsa_gmr083159	TCF3	up
hsa_gmr028429	TCF3	up
hsa_gmr111691	TCF3	up
hsa_gmr119133	TCF3	up
hsa_gmi137105	TCF3	up
hsa_gmr119130	TCF3	up
hsa_gmr091921	TDRKH	up
hsa_gmr099143	TENM2	down
hsa_gmr097186	TFDP2	up
hsa_gmr010368	TGFBR3	up
hsa_gmr028272	TIA1	up
hsa_gmi062133	TIA1	up
hsa_gmr041420	TIA1	up
hsa_gmi062121	TIA1	up
hsa_gmr028257	TIA1	up
hsa_gmr013420	TMEM218	down
hsa_gmr096898	TMEM45A	down
hsa_gmr027732	TPK1	up
hsa_gmr027712	TPK1	up
hsa_gmr027713	TPK1	up
hsa_gmr027715	TPK1	up
hsa_gmr041585	TPK1	up
hsa_gmr027717	TPK1	up
hsa_gmr027719	TPK1	up
hsa_gmr027720	TPK1	up
hsa_gmr027711	TPK1	up
hsa_gmr027723	TPK1	up
hsa_gmr027724	TPK1	up
hsa_gmr027729	TPK1	up
hsa_gmr027730	TPK1	up
hsa_gmr027731	TPK1	up
hsa_gmr027733	TPK1	up
hsa_gmr027721	TPK1	up
hsa_gmr100921	TPK1	up
hsa_gmr077617	TPK1	up
hsa_gmi061852	TPK1	up
hsa_gmr114624	TPK1	up
hsa_gmr114625	TPK1	up

hsa_gmr003479	TPK1	up
hsa_gmr003480	TPK1	up
hsa_gmi061851	TPK1	up
hsa_gmr077622	TPK1	up
hsa_gmr077618	TPK1	up
hsa_gmr077613	TPK1	up
hsa_gmr077614	TPK1	up
hsa_gmr077612	TPK1	up
hsa_gmr064653	TPK1	up
hsa_gmr027706	TPK1	up
hsa_gmr027707	TPK1	up
hsa_gmr027710	TPK1	up
hsa_gmr100914	TPK1	up
hsa_gmr100919	TPK1	up
hsa_gmr100920	TPK1	up
hsa_gmi054939	TPRA1	up
hsa_gmr040066	TREM1	down
hsa_gmr076937	TREM1	down
hsa_gmr076936	TREM1	down
hsa_bmr036264	TRH	up
hsa_bmr092844	TRIM67	up
hsa_gmi128071	TRMT10B	up
hsa_gmr003714	TSEN34	down
hsa_gmr120393	TSGA10	down
hsa_gmr026377	TSGA10	down
hsa_gmr091884	TXNIP	up
hsa_gmr020274	TXNIP	up
hsa_gmr033303	USP13	up
hsa_gmr003431	USP44	down
hsa_gmr048606	VAPA	up
hsa_gmr044463	VCAM1	up
hsa_gmr010927	VCAM1	up
hsa_gmr003790	VCAM1	up
hsa_gmi145979	VIPR1	down
hsa_gmi059110	VTI1A	down
hsa_gmr078677	WHRN	up
hsa_gmr025684	WHRN	up
hsa_gmr084210	WNT7B	down
hsa_gmr085555	XDH	down
hsa_gmr104347	ZFAND4	up
hsa_gmr009579	ZFX	up
hsa_gmr069111	ZNF326	up
hsa_gmr111147	ZNF418	up
hsa_bmr111453	ZNF880	up
